# Blunt Trauma-Induced Chylothorax in Noonan Syndrome: A Rare Clinical Presentation

**DOI:** 10.7759/cureus.104249

**Published:** 2026-02-25

**Authors:** Roxanne Wiblin, Cody Kaiser, Elizabeth Kantzler, Feroze Sidhwa

**Affiliations:** 1 Osteopathic Medicine, Touro University California, Vallejo, USA; 2 Surgery, San Joaquin General Hospital, French Camp, USA; 3 General Surgery/Trauma and Critical Care, San Joaquin General Hospital, French Camp, USA

**Keywords:** blunt thoracic trauma, blunt trauma, chylothorax, noonan, noonan syndrome, right-sided chylothorax

## Abstract

Chylothorax, the accumulation of lymphatic fluid within the pleural cavity, is rarely caused by blunt trauma. We present the case of a 26-year-old male involved in a high-speed motor vehicle collision who sustained a T8 chance fracture and developed progressive right-sided pleural effusion. The patient was managed conservatively with drainage and dietary modification. His clinical history of congenital cardiac surgery, abnormal facies, and prior pleural effusion raised suspicion for Noonan syndrome, which has been associated with lymphatic maldevelopment and predisposition to chylothorax. This case underscores the importance of including chylothorax in the differential diagnosis of pleural effusion following blunt thoracic trauma, particularly in patients with underlying lymphatic abnormalities, and highlights the effectiveness of conservative management in low-output cases.

## Introduction

Chylothorax is a rare outcome of blunt trauma. The thoracic duct is injured, releasing triglyceride-rich chylous fluid into the pleural cavity, which is more common with penetrating or iatrogenic mechanisms of injury, such as a thoracic surgical error [1]. Patients with chylothorax will commonly present with dyspnea and a heavy feeling in their chest. A chest X-ray will show a right-sided pleural effusion, as the thoracic duct ascends on the right side of the thoracic cavity before crossing over at approximately T5, though bilateral effusions are possible. In penetrating injuries, the onset of effusion is rapid. In an iatrogenic injury, the accumulation begins more gradually as reintroduction of oral intake increases [2]. As the patient's body produces chylomicrons from the fat in their diet, chyle production increases, and more fluid is released into the thoracic cavity via the defect in the thoracic duct.

Low output chylothorax is defined as <1 L of fluid output per day and is managed conservatively with drainage and diet modifications, which has been shown to resolve 50% of traumatic chylothoraces [3]. By reducing the fat in the diet, fewer chylomicrons are produced, and less lymph will travel through the lymphatic ducts, allowing for healing. Medium-chain fatty acids are unique in that these lipids are not repackaged into chylomicrons and distributed in chyle and can instead travel directly to the bloodstream via the portal vein.

Chylothorax can be differentiated from other causes of pleural effusion through clinical reasoning and fluid analysis. Pleural aspirate is routinely analyzed for protein content, cell count with differential, bacterial culture, Gram stain, lactate dehydrogenase (LDH), and pH. Initial clinical suspicion can be based on the color and character of the effluent. For example, bloody fluid makes hemothorax more likely, and frank purulence brings empyema to the top of the differential [4]. A triglyceride level of >110 mg/dL and chylomicron presence in chest tube fluid are used to clinically diagnose chylothorax [5], but can be suspected if chest tube fluid has a milky appearance. A potential complication of prolonged chyle leak is malnutrition, due to loss of chylomicrons, which carry lipids and fat-soluble vitamins to the circulatory system. This can lead to prolonged periods of healing, coagulopathies, and longer overall hospital stays [6].

Noonan syndrome is a congenital syndrome associated with short stature and congenital heart disease, as well as characteristic facies and abnormal lymphatic system phenotypes. These patients are often of average intellectual function, and many patients with mild presentations may go their lives without being diagnosed [7].

This study aims to review common etiologies, diagnostic factors, and management of chylothorax in the context of a unique mechanism of injury and patient presentation.

## Case presentation

A 26-year-old male was the restrained driver in a high-speed motor vehicle crash. The patient was brought in by ambulance, complaining of back and neck pain. On T2-weighted MRI, a chance T8 fracture and right pleural effusion were discovered. Also evidenced in the scan was a history of prior cardiac surgery, although the reason was unclear.

**Figure 1:**
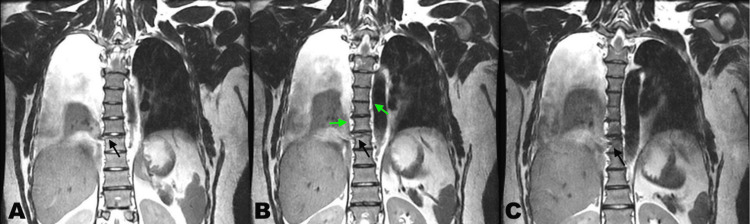
T2-weighted MRI demonstrating T8 chance fracture with associated thoracic duct rupture (black arrows) and ductal duplication (green arrows). Panels A-C represent three contiguous coronal T2-weighted slices arranged from anterior (A) to posterior (C). Black arrows indicate disruption of the thoracic duct, and green arrows demonstrate duplicated thoracic ducts present on both sides of the spine. A right-sided pleural effusion is present in all panels. The thoracic duct rupture and duplication are most clearly visualized in panel B. Images were obtained on a 3.0-T MRI scanner with fat suppression.

On hospital day two, the patient became tachypneic and diaphoretic and reported pain on inspiration. A stat CT showed a worsened right pleural effusion, and the decision was made to place a pigtail catheter and attach it to suction, yielding 1,800 mL of serosanguinous fluid. On hospital day three, it was noted that the fluid in the pleur-evac was topped by a milky white layer, which raised suspicion for chylothorax. The fluid was analyzed and was positive for chylomicrons and yielded a triglyceride level of 798 mg/dL, which exceeds the diagnostic standard of 110 mg/dL for chylothorax. The patient was sent for an MRI to evaluate for potential sources of the leak. A right-sided thoracic duct rupture was discovered, just lateral to the site of the spinal fracture (Figure 1).

**Figure 2:**
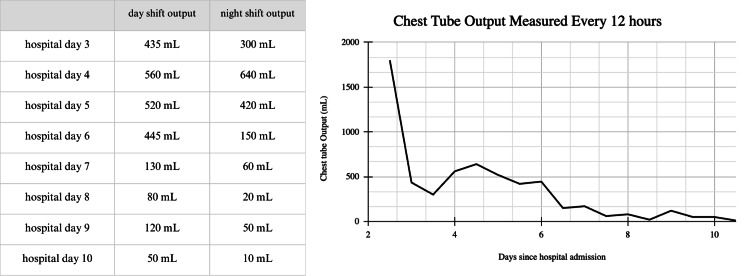
Fluid volume collected from the pigtail catheter following an initial drainage of 1,800 mL on hospital day two. Output was subsequently recorded every 12 hours at shift change.

After confirming the diagnosis, the patient was managed conservatively by modifying his diet to low-fat with medium-chain fatty acids, along with keeping his chest tube to suction. His chest tube output steadily decreased each day of his hospital stay, reduced to water seal on hospital day seven, and removed on hospital day 10 (Figure 2).

## Discussion

This patient's cardiac history included a double switch procedure as an infant for L-transposition of the great arteries. Combining this with a history of childhood pleural effusion, his abnormal facies and body habitus (widely spaced eyes, neck skin webbing, short stature), and confirmed chylothorax led us to believe that he could have this congenital syndrome. In Noonan syndrome, the *RAS *and *MAPK *genes are mutated, leading to overexpression of ERK1 and 2, which are implicated in lymphatic development in the embryo [8]. The phenotypical presentation is varied, but studies have shown cases of retrograde lymphatic flow, cisterna chyli enlargement, lymphatic duct duplication, and, most commonly, lymphedema. Spontaneous or refractory chylothorax has been observed in patients with Noonan syndrome, but are usually only observed in neonates [9,10]. An existing Noonan syndrome diagnosis might have led us to consider chylothorax earlier in this patient's course of treatment.

This case is unique in that only 0.2-3% of chylothoraces observed are due to blunt trauma [11], as a majority are iatrogenic surgical injuries or due to penetrating wounds. A contributing factor in this case was the mechanism of injury. Being struck from behind in a motor vehicle collision causes hyperextension of the spine, which can lead to stretching of the lymphatic duct that lies anterior to the spine. This longitudinal stretching and shear stress from the diaphragmatic crus can lead to rupture [12]. In addition, due to the patient's suspected Noonan syndrome, his abnormal lymphatic phenotype could have contributed to the patient developing a chylous effusion.

Our patient responded well to conservative treatment, though in refractory cases of chylothorax, treatment escalates to more invasive methods, including percutaneous thoracic duct embolization, lymphangiography with poppy seed oil, or surgical repair [5]. Other pharmacotherapy options include midodrine, which can cause lymphatic constriction, and octreotide, a somatostatin analogue that works to inhibit lymphatic fluid excretion [6]. Low-output chyle leaks like this one typically resolve between three days and two weeks with conservative management.

Chylothorax should be on the differential diagnosis for pleural effusion, especially in the case of “fat capping” of chest tube aspirate. It should also be considered in cases of hyperextension injury. This is especially pertinent in patients with abnormal lymphatic circulation.

## Conclusions

We report a case of traumatic chylothorax in a patient with suspected Noonan syndrome. This case is unique in both the mechanism of injury and the underlying congenital lymphatic abnormalities. Chylothorax should be on the differential diagnosis for Noonan syndrome patients with pleural effusion, especially in the case of “fat capping” of chest tube aspirate. It should also be considered in cases of hyperextension injury. Further study is needed to determine the risk of trauma-related lymphatic injury in Noonan syndrome. Early recognition and conservative management in this case led to a favorable outcome without the need for surgical intervention.
